# Plasma Long Noncoding RNA *LeXis* is a Potential Diagnostic Marker for Non-Alcoholic Steatohepatitis

**DOI:** 10.3390/life10100230

**Published:** 2020-10-03

**Authors:** Jung Gil Park, Gyeonghwa Kim, Se Young Jang, Yu Rim Lee, Eunhye Lee, Hye Won Lee, Man-Hoon Han, Jae Min Chun, Young Seok Han, Jun Sik Yoon, Min Kyu Kang, Young Oh Kweon, Won Young Tak, Soo Young Park, Keun Hur

**Affiliations:** 1 Department of Internal Medicine, College of Medicine, Yeungnam University, Daegu 42415, Korea; gsnrs@naver.com (J.G.P.); kmggood111@naver.com (M.K.K.); 2 Department of Biochemistry and Cell Biology, School of Medicine, Kyungpook National University, Daegu 41944, Korea or aoet111@gmail.com (G.K.); eunhye7832@naver.com (E.L.); 3 Department of Internal Medicine, School of Medicine, Kyungpook National University, Kyungpook National University Hospital, Daegu 41944, Korea; magnolia1103@naver.com (S.Y.J.); deblue00@naver.com (Y.R.L.); yokweon@knu.ac.kr (Y.O.K.); wytak@knu.ac.kr (W.Y.T.); 4 Department of Pathology, Dongsan Medical Center, School of Medicine, Keimyung University, Daegu 42601, Korea; hoongirl82@naver.com; 5 Department of Pathology, School of Medicine, Kyungpook National University, Kyungpook National University Hospital, Daegu 41944, Korea; one-many@hanmail.net; 6 Department of Surgery, School of Medicine, Kyungpook National University, Kyungpook National University Hospital, Daegu 41944, Korea; cjmdr@knu.ac.kr (J.M.C.); gshyskhk@hanmail.net (Y.S.H.); 7 Department of Internal Medicine, Busan Paik Hospital, Inje University College of Medicine, Busan 74392, Korea; yojusi@naver.com

**Keywords:** biomarker, liver fibrosis, long noncoding RNA *LeXis*, non-alcoholic fatty liver disease, non-alcoholic steatohepatitis, untranslated RNA

## Abstract

Non-invasive diagnostic markers are needed to ease the diagnosis of non-alcoholic steatohepatitis (NASH) among patients with non-alcoholic fatty liver disease (NAFLD). The long noncoding RNA (lncRNA) *LeXis* is related to cholesterol metabolism and hepatic steatosis in mice, and its batch genome conversion in humans is TCONS_00016452. Here, we aimed to evaluate the potential of lncRNA *LeXis* as a non-invasive diagnostic marker for NASH. We analyzed a total of 44 NAFLD patients whose diagnosis was confirmed by a pathologist through analysis of a percutaneous liver biopsy. The expression of *LeXis* in the plasma of NAFLD patients with and without NASH was compared using quantitative real-time polymerase chain reaction. The expression of plasma *LeXis* was significantly higher in patients with NASH than in those with NAFL (8.2 (5.0–14.9); 4.6 (4.0–6.6), *p* = 0.025). The area under the receiver operating characteristic curve was 0.743 (95% CI 0.590–0.895, *p* < 0.001), and a sensitivity of 54.3% and specificity of 100% could be achieved for NASH diagnosis. Low *LeXis* was independently associated with NASH diagnosis in patients with NAFLD (*p* = 0.0349, odds ratio = 22.19 (5% CI, 1.25–395.22)). Therefore, circulating lncRNA *LeXis* could be a potential non-invasive diagnostic biomarker for NASH.

## 1. Introduction

Non-alcoholic fatty liver disease (NAFLD) is the most common chronic liver disease, affecting one-fourth of the population in western countries [1]. NAFLD prevalence has increased concomitantly with the increase in the number of obese individuals, thus representing elevated healthcare costs over time [2]. In the initial stages, NAFLD patients show excessive accumulation of fat in the liver without inflammation, which may later progress to an inflammation process, with cell damage and fibrosis [3]. The pathology of NAFLD ranges widely, from non-alcoholic fatty liver (NAFL) without liver cell injury to non-alcoholic steatohepatitis (NASH), leading to advanced fibrosis and cirrhosis, which can ultimately cause hepatocellular carcinoma [4]. Owing to its variable clinical progression, the identification of patients with poor prognosis among those with NAFLD is difficult. A recent study suggested that the presence of advanced fibrosis is a useful factor in predicting overall mortality in NAFLD patients; however, NASH is important for the pathogenesis of liver fibrosis [1,5,6,7]. Nevertheless, liver biopsy is the only confirmatory test available for the diagnosis of NASH and to ascertain NAFLD grading and staging. Non-invasive approaches for assessing liver fibrosis in NAFLD using demographic characteristics and biochemical markers were proposed in several studies [8], but no reliable biomarker to diagnose NASH has yet been identified. Thus, the identification of a diagnostic marker for the diagnosis of NASH via a non-invasive testing is necessary.

Long noncoding RNAs (lncRNAs) are a type of transcribed RNA molecule, longer than 200 nucleotides, that do not encode proteins [9]. They are known to be important regulators of gene expression and act via diverse mechanisms, including chromatin modification involving the conformation of the nuclear domain, the activation of transcriptional enhancers, and interference with transcriptional machinery [10,11]. Furthermore, some lncRNAs are involved in post-transcriptional processes including the regulation of splicing, the decay of mRNA and miRNA, and protein translation and stability [11]. Liver-expressed liver X receptors (LXR) induced sequence (*LeXis*) is a lncRNA that was recently identified in mice livers and was found to regulate lipid metabolism hemostasis in the liver and plasma by interacting with the ribonucleoprotein RALY [12]. Chronic inactivation of *LeXis* increases the hepatic cholesterol content without inflammation or endoplasmic reticulum (ER) stress. However, to date, the hepatic and plasma expression of *LeXis* in patients with NAFLD has not been not evaluated.

In the present study, we analyzed the expression of the lncRNA *LeXis* in both the liver tissue and plasma of patients with NAFLD by evaluating lncRNA TCONS_00016452, the corresponding human batch genome conversion of mouse lncRNA *LeXis*.

## 2. Methods

### 2.1. Patient Samples

Between January 2014 and June 2016, a total of 44 liver tissue biopsies and plasma samples were obtained from patients with pathologically confirmed NAFLD at Kyungpook National University Hospital. All tissue samples were obtained using ultrasound-guided percutaneous liver biopsy on the same day that biochemical parameters and a plasma sample were obtained from the patients, to minimize possible confounding factors. This study was approved by the ethical committees of the institute (KNUH 2018-05-025-003), and written informed consent was obtained from all patients.

### 2.2. NAFLD Diagnois and Pathological Evaluation

**Figure 1:**
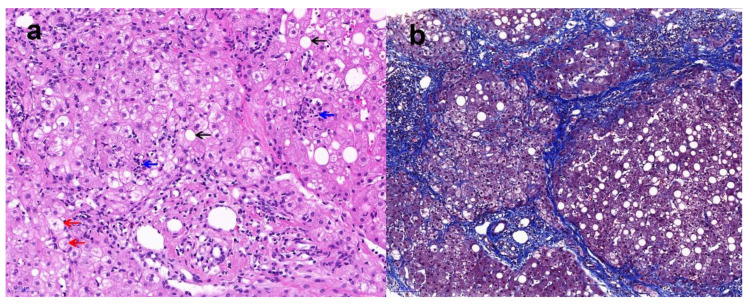
(**a**) Steatosis (black arrow), lobular inflammation (blue arrow), and ballooning degeneration (red arrow) were scored on 1–3, 0–3, and 0–2 scale (hematoxylin and eosin staining; amplification, 200×). (**b**) The fibrosis stage was determined using the Kleiner scoring system as F0–F4 (trichrome staining; amplification, 100×).

The diagnosis of NAFLD was made by a pathologist via the identification of at least 5% hepatic fat accumulation in pathology without excessive alcohol intake (men >140 g/week; women >70 g/week). Any other possible causes of chronic liver disease were excluded via analysis of serologic markers for viral hepatitis and the evaluation of medical records. All specimens were reviewed by a single experienced pathologist to avoid inter-observer variability. The diagnosis of NASH was made according to the current guidelines of the American Association for the Study of Liver Diseases [13]. Fibrosis staging and NAFLD activity score (NAS) were assessed according to recommendations of the Pathology Committee of the NASH Clinical Research Network (Figure 1) [14]. Severe NAFLD was defined as NAS above 4.

### 2.3. RNA Extraction

For total RNA extraction from clinical tissues, 1 mL of QIAzol Lysis Reagent (Qiagen, Hilden, Germany) was added to 100 mg of tissues. The tissue samples were homogenized and incubated for 5 min at room temperature (RT). Next, 0.2 mL chloroform was added to each tube, followed by vigorous stirring for 30 sec and additional 5-min incubation at RT. The samples were centrifuged at 12,000× *g* for 15 min at 4 °C. The upper aqueous phase was transferred to a new tube that was mixed by vortexing after added 0.5 mL isopropanol. After 10 min incubation at RT, the tube was centrifuged again at 12,000× *g* for 10 min at 4 °C, and the supernatant was discarded. The pellet was washed with 75% ethanol and centrifuged at 7500× *g* for 5 min at 4 °C. The pellet was air dried and resuspended in 20 μL of RNase-free water, and then stored at −80 °C until use.

The plasma sample was separated from whole peripheral blood by centrifugation at 3000 rpm for 10 min at 4 °C. Next, a miRNeasy Serum/Plasma Kit (Cat No./ID: 217204, Qiagen) was used to extract total RNA from the plasma, as described previously [15]. Briefly, 200 μL of plasma sample was mixed with 60 μL of Buffer RPL (Qiagen) and incubated for 3 min at RT. The sample was mixed with spike-in control (1.6 × 10^8^ copies/μL of cel-miR-39) and 20 μL of Buffer RPP; and the tube was mixed vigorously for 30 sec and incubated for 3 min at RT. After centrifuging at 12,000× *g* for 3 min at RT, the supernatant was transferred to a new tube, which was then mixed by vortexing after adding 0.2 mL of isopropanol. The mixture solution was transferred onto the column and was centrifuged at 8000× *g* for 15 min. After removing the flow-through, 700 μL of Buffer RWT (Qiagen) was added, and the column was centrifuged again at 8000× *g* for 15 min. This process was repeated with 500 μL of Buffer RPE (Qiagen) and then with 500 μL of 80% ethanol; the solution was then centrifuged at 10,000× *g* for 2 min. Lastly, the RNA captured in the column filter was eluted in 20 μL of RNase-free water.

The quantity and quality of the total extracted RNA was evaluated on a NanoDrop ND-1000 spectrophotometer (NanoDrop Technologies, Wilmington, DE, USA) was used.

### 2.4. Quantitative Real-Time PCR

For reverse transcription, a High-Capacity cDNA Reverse Transcription Kit (Thermo Fisher Scientific Inc., Waltham, MA, USA) was used with 800 µg of total RNA according to the manufacturer’s protocol for tissue samples. The protocol was performed with 1 µL of the solution, which involved the dilution of the resulting cDNA with 80 µL of distilled water. For reverse transcription of the plasma samples, 500 µg of total RNA was used which was diluted with 80 µL of distilled water. The protocol was performed with 4 µL of this diluted cDNA. Each sample was processed in triplicate via quantitative real-time PCR using SYBR Green PCR Master Mix (Thermo Fisher Scientific Inc.). The primer sequences of *LeXis* were used as follows: TCONS-00016452 (forward), 5′–TCACATCTCCTCCGTTTCAGAG–3′, and TCONS-00016452 (reverse), 5′–GTT ATGCGTCATGCCAAAGAAATC–3′. The PCR conditions were as follows: 2 min at 50 °C and 10 min at 95 °C, followed by 45 cycles of 15 s at 95 °C and 60 s at 60 °C. *GAPDH* (Glyceraldehyde-3-Phosphate Dehydrogenase) was used as the endogenous control, and the relative expression of *LeXis* was determined via the 2^−ΔΔCt^ method.

### 2.5. Statistical Analysis

All continuous data are expressed as the means ± standard deviations or medians with interquartile ranges after testing for normality. All categorical data are expressed as numbers with percentages. Significant differences between patients were analyzed using the chi-square test, Student’s *t*-test, Mann-Whitney *U* test, or Fisher’s exact test. The analysis of the correlation between *LeXis* and steatosis was performed using the Spearman’s correlation coefficient. Receiver operating characteristic (ROC) analysis was conducted to assess the diagnostic performance of *LeXis* for NASH. All statistical analyses were performed using the R software (version 3.2.2; R Foundation for Statistical Computing, Vienna, Austria). A *p* value less than 0.05 was considered statistically significant.

## 3. Results

### 3.1. Hepatic lncRNA LeXis is Downregulated in Patients with Severe Hepatic Steatosis

**Figure 2:**
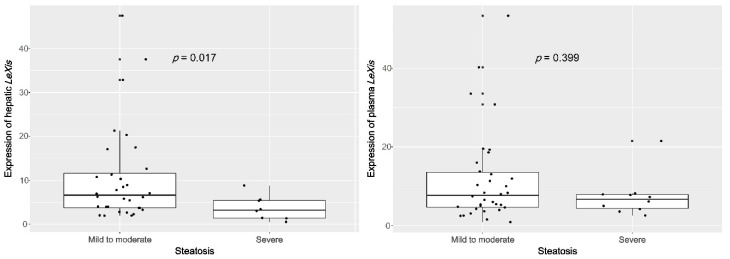
The expression of hepatic *LeXis* is significantly upregulated in patients with severe steatosis compared with those without.

**Figure 3:**
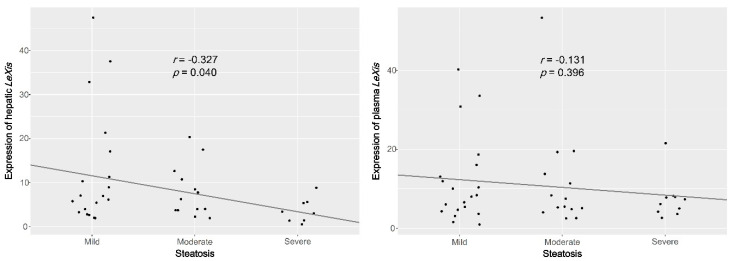
The degree of steatosis is negatively correlated with hepatic lncRNA *LeXis* levels but not with plasma lncRNA *LeXis* levels.

As reported previously, the expression of hepatic *LeXis* was significantly downregulated in patients with severe steatosis compared with that in patients with mild-to-moderate steatosis (S = 1–2: 6.6 (3.7–12.0); S = 3: 3.2 (1.4–5.5); *p* = 0.017). However, the expression of plasma *LeXis* was not significantly different between the patient groups (S = 1–2: 7.7 (4.6–13.7), S = 3: 6.7 (4.2–7.9), *p* = 0.399; Figure 2). In addition, the degree of steatosis was negatively correlated with hepatic *LeXis* levels but not with plasma *LeXis* levels. (tissue, *r* = −0.327, *p* = 0.040; plasma, *r* = −0.131, *p* = 0.396; Figure 3).

### 3.2. Plasma lncRNA LeXis Levels are Increased in Patients with NASH

**Figure 4:**
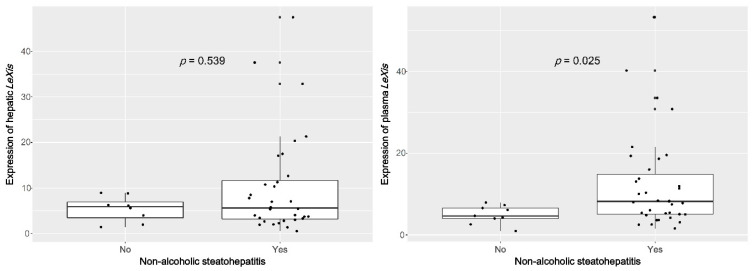
The expression of plasma *LeXis* is significantly upregulated in patients with NASH compared with those without.

**Figure A1:**
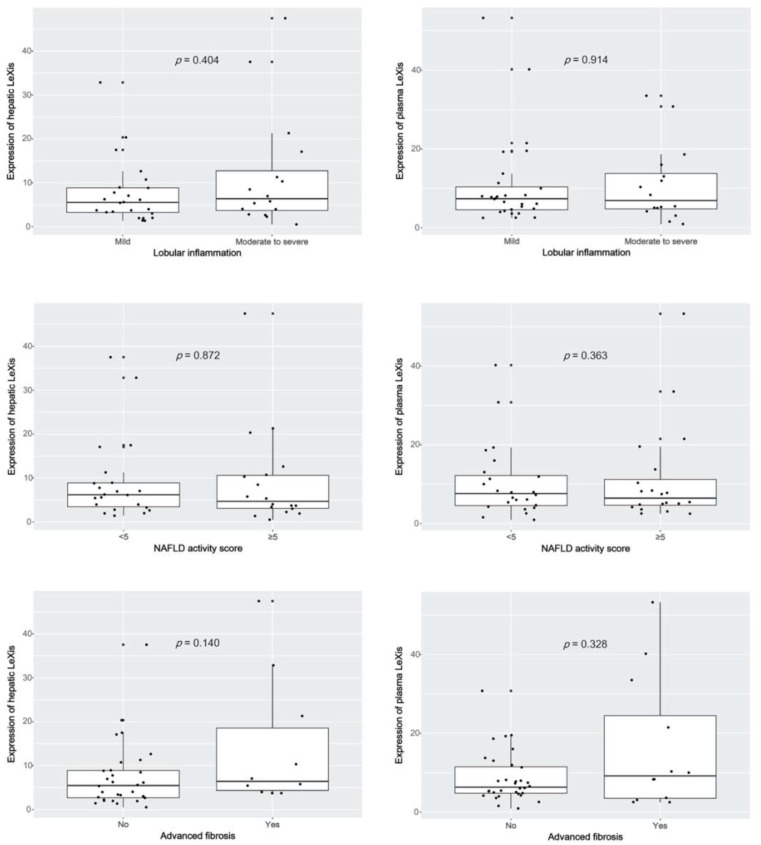
Expression of tissue and plasma *LeXis* according to the degree of inflammation, severity of NASH, and stage of fibrosis in patients with NAFLD.

**Figure A2:**
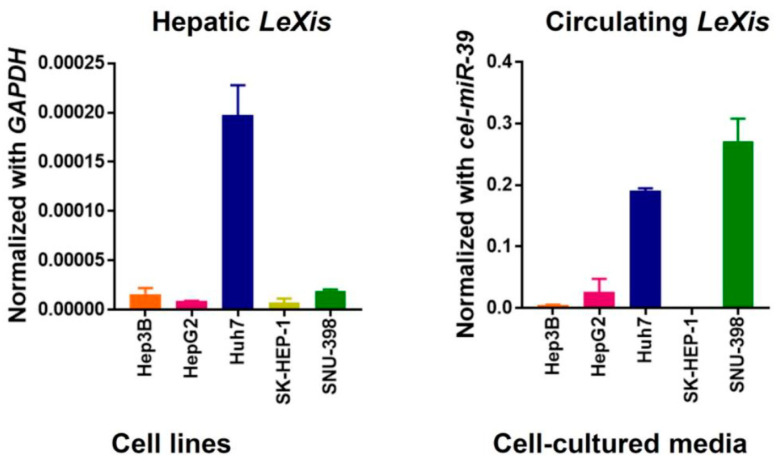
Expression of *LeXis* in hepatocellular carcinoma cell lines and their cultured media.

In the pathology results, one-half of the NASH patients showed severe NAFLD (NAS ≥ 5), and significant fibrosis (F ≥ 2), whereas none of the non-alcoholic fatty liver (NAFL) patients exhibited high NASH or significant fibrosis (Table 1). The patients with NASH were significantly older (*p* = 0.020) and showed lower serum albumin levels (*p* = 0.043) than those without NASH. None of the other patient characteristics were significantly different between these groups and were thus not associated with the diagnosis of NASH. The expression of *LeXis* did not differ significantly with regard to the degree of inflammation, NASH severity, or stage of liver fibrosis in both tissue and plasma samples (Figure A1). Contrary to our expectation, the expression of plasma *LeXis* was significantly upregulated in patients with NASH compared with that in those without (4.6 (4.0–6.6) vs. 8.2 (5.0–14.9), *p* = 0.025; Figure 4). However, hepatic *LeXis* did not significantly differ between patients with or without NASH (5.9 (3.0–7.5) vs. 5.6 (3.2–12.0), *p* = 0.539). To confirm the stability of plasma *LeXis*, we evaluated the expression of *LeXis* in cell-cultured media. Among the five hepatocellular carcinoma cell lines tested, we could detect the expression of *LeXis* in four cultured media (Figure A2).

### 3.3. Predictive Cut-off Value and Diagnostic Performance of Plasma LeXis Levels for Diagnosing NASH.

**Figure 5:**
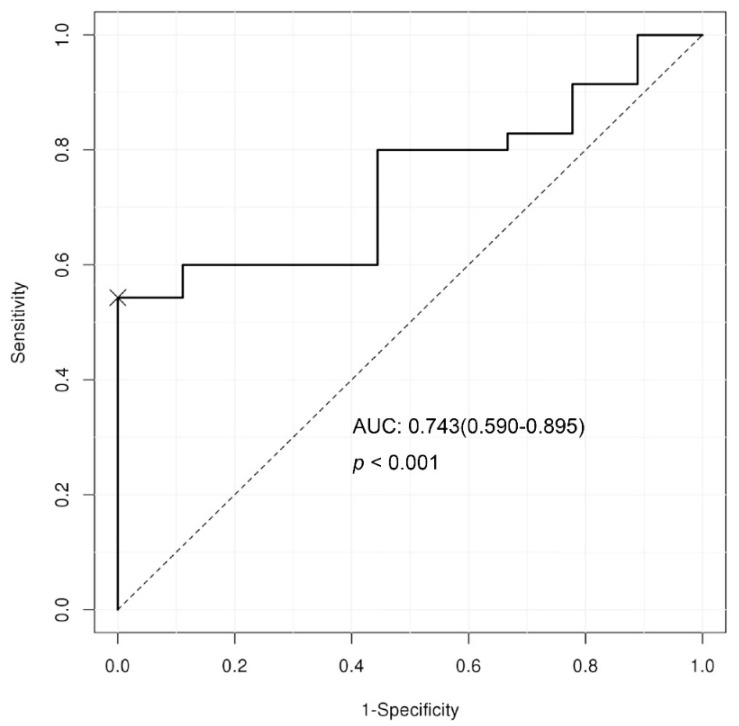
The area under the ROC curve of the plasma *LeXis* for diagnosing NASH was 0.743.

The area under the ROC curve of the plasma *LeXis* for diagnosing NASH was 0.743 (95% CI 0.59–0.895, *p* < 0.001; Figure 5). The maximal sum of the sensitivity (54.3%) and specificity (100%) for diagnosing NASH could be achieved at a relative expression level of 7.92. Thus, all the patients with plasma *LeXis* levels above 7.92 were diagnosed with NASH. In addition, high plasma levels of *LeXis*, as defined by the cut-off value, were independently associated with NASH diagnosis in patients with NAFLD. (*p* = 0.0349, odds ratio = 22.19 (95% CI, 1.25–395.22); Table 2).

## 4. Discussion

In this study, we demonstrated that hepatic *LeXis* is negatively correlated with steatosis, and that patients with NASH have increased expression of plasma *LeXis*. In addition, plasma *LeXis* was independently associated with NASH with an acceptable diagnostic performance. The activation of LXR through pharmacologic intervention or a western diet induces the overexpression of hepatic *LeXis*. The interaction between *LeXis* and RALY inhibits the ability of the protein to bind to the cholesterol biosynthesis gene in the liver, which ameliorates hepatic steatosis. As shown in a previous report, downregulation of hepatic *LeXis* induces the vulnerable state of hepatic steatosis by altering the lipid metabolism. However, downregulated *LeXis* does not affect ER stress and inflammation, which are important pathological features of NASH [12]. Herein, we found that hepatic *LeXis* was downregulated as hepatic steatosis progressed to severe grades, which was not related to inflammation and ballooning, in accordance with previous reports. However, plasma *LeXis* was found not to reflect steatosis and inflammation, which are related to the ballooning degeneration of hepatocytes. As ballooned hepatocytes are considered a hallmark of steatohepatitis, decreased keratin 8/18 immunostaining in the cytoplasm was proposed as an objective marker for specifically identifying ballooned hepatocytes in patients with NAFLD [16]. Based on this study, the reciprocal elevation of plasma caspase-generated cytokeratin-18 fragments (CK-18) was proposed as a promising non-invasive biomarker for NASH diagnosis. However, a single CK-18 test revealed its lack of diagnostic potential for NASH [17,18]. In the present study, plasma *LeXis* was significantly upregulated in NASH and was also found to be a specific biomarker for NASH diagnosis in patients with NAFLD, regardless of other clinical factors such as hypertension and diabetes, or the levels of aminotransferase, creatinine, and platelets. In addition, plasma LeXis was also found to be independent of other pathological conditions, including steatosis, lobular inflammation, and fibrosis stage. Therefore, we believe that hepatocytes apoptosis followed by ballooning degeneration could induce the upregulation of plasma *LeXis*.

*LeXis* has been reported to promote the growth of osteosarcoma through the upregulation of CTNNB1 expression [19]. However, to date circulating *LeXis* has not been studied yet in patients with NAFLD or those with cancer.

Our study has certain limitations. First, although significant differences were shown, functional assessment of plasma *LeXis* in NASH was not performed. Nevertheless, the functional analysis of hepatic *LeXis* described in this study was consistent with the previous mouse data. Second, the number of patients with NAFL included in the study was relatively small. and the evaluated cohort lacked healthy controls. Third, no external validation was performed on the diagnostic performance of plasma *LeXis* for NASH. Nonetheless, this is the first study to suggest plasma *LeXis* as a potential diagnostic biomarker for NASH. We confirmed that hepatic *LeXis* is not associated with NASH, as previously described [12], whereas plasma *LeXis* was found to be independently associated with NASH. In the present study, however, the underlying mechanism of these findings could not be determined; thus, further evaluation is required in future investigations.

In conclusion, the overexpression of plasma lncRNA *Lexis* is associated with the ballooning degeneration of hepatocyte in patients with NAFLD. Moreover, circulating lncRNA *LeXis* could represent a useful non-invasive diagnostic biomarker for NASH.

## Figures and Tables

**Table 1:** Characteristics of patients.

Characteristics	Non-Alcoholic Fatty Liver Disease (NAFL) (*n* = 9)	Non-Alcoholic Steatohepatitis (NASH) (*n* = 35)	*p* Value
Male	2 (22.2)	20 (57.1)	0.135
Age, year	38.3 ± 12.0	51.9 ± 15.5	0.020 *
Steatosis			0.144
5-33	4 (44.4)	16 (45.7)	
>33–66	1 (11.1)	13 (37.1)	
>66%	4 (44.4)	6 (17.1)	
Lobular inflammation			0.197
<2 foci per 200× field	8 (88.9)	20 (57.1)	
2–4 foci per 200× field	1 (11.1)	11 (31.4)	
>4 foci per 200× field	0 (0.0)	4 (11.4)	
Ballooning			0.000 *
None	9 (100.0)	0 (0.0)	
Few ballooned cells	0 (0.0)	20 (57.1)	
Many cells/prominent ballooning	0 (0.0)	15 (42.9)	
Advanced fibrosis	0 (0.0)	12 (34.3)	0.101
NAS ≥ 5	0 (0.0)	20 (57.1)	0.007 *
Weight, kg	83.3 ± 20.3	71.7 ± 11.6	0.182
BMI, kg/m^2^	31.0 (26.8–33.1)	27.1 (25.9–30.4)	0.348
Hypertension	4 (44.4)	17 (48.6)	1.000
Diabetes	1 (11.1)	15 (42.9)	0.168
Platelets, × 10^3^/mm	240.4 ± 51.3	219.8 ± 71.5	0.426
AST, IU/L	57.0 (43.0–95.0)	78.0 (46.5–106.5)	0.211
ALT, IU/L	90.0 (68.0–147.0)	98.0 (66.5–120.5)	0.816
Bilirubin, mg/dL	0.7 (0.5–0.8)	0.6 (0.4–0.6)	0.119
Albumin, g/dL	4.7 ± 0.3	4.5 ± 0.3	0.043 *
γ-GTP, mg/dL	55.0 (43.0–95.0)	77.0 (55.0–124.0)	0.366
Creatinine, mg/dL	0.9 (0.8–1.0)	0.8(0.6–0.9)	0.134
Fasting blood glucose, mg/dL	107.0 (102.0–113.5)	117.5 (104.0–136.0)	0.133
Total cholesterol, mg/dL	179.0 (162.5–188.5)	177.0 (159.0–210.0)	0.835
HDL, mg/dL	47.5 (35.5–57.5)	41.5 (33.0–50.0)	0.441
LDL, mg/dL	117.0 ± 25.7	117.7 ± 38.3	0.963
Triglyceride, mg/dL	161.5 (115.5–194.5)	142.5 (112.0–213.0)	0.946

Values are presented as the mean ± standard deviation, median (interquartile ranges) or number (%). ALT, alanine aminotransferase; AST, aspartate aminotransferase; BMI, body mass index; GTP, guanosine triphosphate; HDL, high-density lipoprotein; LDL, low-density lipoprotein; NAS, non-alcoholic fatty liver disease activity score. * *p* < 0.05 was considered statistically significant.

**Table 2:** Factors associated with risk for non-alcoholic steatohepatitis.

Variable	Univariate Analysis		Multivariate Analysis	
	Odds Ratio	*p* Value	Odds Ratio	*p* Value
Sex	4.67 (0.85–25.75)	0.0771		
Age	1.06 (1.01–1.12)	0.0298		
Hypertension	1.18 (0.27–5.15)	0.8251		
Diabetes	6.00 (0.68–53.29)	0.1078		
Platelets	1.00 (1.00–1.00)	0.4175		
AST	1.02 (0.99–1.05)	0.1310	1.04 (0.99–1.08)	0.136
ALT	1.00 (0.99–1.01)	0.8114		
Bilirubin	0.17 (0.02–1.78)	0.1385	0.02 (0.00–0.68)	0.029 *
Albumin	0.07 (0.00–1.09)	0.0574		
Creatinine	0.10 (0.00–4.10)	0.2263		
High plasma *LeXis*	9.50 (1.07–84.25)	0.0432	22.19 (1.25–395.22)	0.035 *

ALT, alanine aminotransferase; AST, aspartate aminotransferase; * *p* < 0.05 was considered statistically significant.
